# Morphological, Mechanical and Gas Penetration Properties of Elastomer Composites with Hybrid Fillers

**DOI:** 10.3390/polym14194043

**Published:** 2022-09-27

**Authors:** Tuba Evgin, Matej Mičušík, Peter Machata, Hamed Peidayesh, Jozef Preťo, Mária Omastová

**Affiliations:** 1 Polymer Institute of the Slovak Academy of Sciences, 845 41 Bratislava, Slovakia; 2 Mechanical Engineering Department, Engineering Faculty, Dokuz Eylul University, Izmir 35397, Turkey; 3 Vipo, a.s., Generála Svobodu 1069/4, 958 01 Partizánske, Slovakia

**Keywords:** polymer composites, ethylene–propylene–diene monomer (EPDM), graphene nanoplatelets (GnPs), carbon black (CB), mechanical properties, dynamic mechanical analysis (DMA), gas penetration

## Abstract

Ethylene–propylene–diene monomer (EPDM)-based composites including four different types of graphene nanoplatelets (GnPs) were prepared to evaluate the size effects of GnPs in terms of both specific surface area and lateral size on the morphological, mechanical, and viscoelastic properties, swelling ratio, crosslink density, and oxygen permeability. EPDM-based hybrid composites with GnPs and carbon black (CB) fillers were prepared, with the concentrations of 20 and 50 phr of CB and GnPs up to 7 phr. All samples were prepared using the melt mixing method, followed by compression molding. The specific surface area of GnPs is a more important key factor for mechanical and viscoelastic properties than its lateral size. The presence of GnPs leads to a decrease in the swelling ratio and oxygen permeability of the matrix while an increase in the crosslinking density. For a given specific surface area of GnPs (170 m^2^/g) and the same thickness (5 nm), the optimum lateral size for mechanical properties, swelling ratio, and crosslinking density is about 30 µm. There is a distinct synergic effect on the mentioned properties when hybrid fillers are used. For hybrid composites, the optimum total and each filler concentration are found to be important for achieving the best performance in terms of mechanical properties, swelling ratio, and crosslink density.

## 1. Introduction

A number of studies on rubber polymer composites have been carried out in recent years [1,2,3,4,5,6,7,8,9,10,11,12,13,14,15,16,17,18,19,20,21,22,23,24,25,26,27,28,29,30]. It is reported that the enhancement in overall attributes of rubber composites depends on the diverse parameters such as the aspect ratio and size of nanofillers, their location in the rubber matrix, the dispersion degree of nanofillers, and the adhesion degree with the elastomer chains [11]. However, many issues are still not well explained and understood. It is known that rubber composites are multi-component and complicated systems with crosslinking structures. Because of this, understanding the relationship between properties and microstructure is difficult [5].

The ethylene–propylene–diene monomer (EPDM) has been gaining increasing attention especially due to its high thermal and thermo-oxidative stability, compared to other high-volume rubbers. Further attractive features are the high resistance to ultraviolet (UV) light, oxygen, fatigue, weather, and moisture resistance [16,31]. Moreover, EPDM is widely used in many applications due to its ability to be reinforced with high nanofiller loading [9,13]. EPDM composites with nanofillers (metal nanofillers, carbon black (CB), graphene and carbon nanotubes (CNTs), etc.) are used for different applications including actuators, EMI shielding, pressure-sensitive switches, microwave absorption, biosensors, touch pads, conductive gaskets, and many others [16,31]. Considering the reinforcing fillers, CB is the most frequently used nanofiller for rubber matrix because of providing good reinforcement to rubber [9,32]. However, a relatively large amount of CB is required to achieve the desired properties of the composites [31] resulting in a significant increase in the melt viscosity, making the melt processing of polymer composite more difficult [10]. Although CB remains the dominant filler for the elastomer composites, using other fillers for EPDM composites are investigated. Among these, graphene becomes a promising nanofiller due to its high mechanical (intrinsic strength 130 GPa and Young’s modulus 1 TPa), electrical, and thermal properties [22,30]. Graphene affects positively the design of the composites with high performance at low nanofiller loading due to its honeycomb structures [31]. As result, the researchers have reported that graphene and its derivates are promising nanofillers since they provide not only structural reinforcement but also enhance the thermal and electrical conductivity of elastomers [4]. Therefore, the EPDM composites reinforced with graphene have attracted interest.

The dispersion of nanofillers in a matrix is crucial in rubber reinforcement. However, graphene tends to agglomerate leading to a decrease in the surface energy of nanofillers due to Van der Waals interactions [15,33]. The application of hybrid nanofillers is an effective and low-cost strategy. The carbon nanofillers with different geometries are suitable for forming co-supporting networks at a special mass ratio when the hybrid carbon-based nanofillers are uniformly dispersed in the matrix [34]. The reason for improved properties consists of the various geometric shapes, aspect ratios, and dispersion characteristics of two fillers offering a unique synergy [35], i.e., the properties’ enhancement is different for the polymer composites with various nanofillers [36]. For example, while CNTs show better performance for enhancing the electrical and thermal conductivities of polymers [36,37], graphene nanoplatelets (GnPs) indicate certain additional benefits such as possessing higher gas permeation resistance and lower production cost due to their platelet structure as compared to the other types of carbon nanofillers. Moreover, a combination of more than one type of carbon-based nanofiller with different dimensions can be used to prepare polymer nanocomposites such as 0D atomic clusters (e.g., fullerenes, nano-carbon black), 1D rod-like nanofiller (e.g., CNTs, carbon fiber), and 2D platelet-like nanofiller (e.g., graphene, GnPs) [38]. The use of two geometrically different nanofiller materials, such as multiwalled carbon nanotubes (MWCNTs) and GnPs or expanded graphite (EG) and CB or CB and carbon nanofibers (CNFs), could provide the formation of a co-supporting network of the two nanofiller materials enabling a reduction in the amount of total filler material [35,39]. These layered structures of filler materials create uniform reinforcing pathways in the polymer nanocomposites at a relatively low concertation of filler materials. Hereby, these polymer nanocomposites also exhibit good mechanical properties. It is known that the main objective of carbon-based polymer nanocomposite preparation is to create material with a high thermal and/or electrical conductivity without destroying the mechanical properties or possibility with an enhancement in the mechanical properties [35].

Another obstacle in a graphene-wide application is its relatively high cost. Therefore, GnPs, layered graphene nanocrystals in the platelets structure bonded by van der Waals forces, are an inexpensive alternative because of their interesting properties and possibility of mass fabrication at a lower cost [40,41]. It is known that the GnPs’ properties are similar to the graphene properties. GnPs can display superior mechanical properties (Modulus of Young: 208–650 GPa) and high electrical conductivity (10^3^ S/m) [36]. Due to the unique graphitized plane structure, high in-plane conductivity, and more importantly the low production cost, GnPs seem to be a promising additive for reinforcing the polymer nanocomposites. The GnPs’ planar structure and the high surface area provide high interface interactions with the polymer matrix so long as the stacking of graphene layers is prevented, resulting in desirable enhancements of functional and mechanical properties for the polymers as reported. Notwithstanding, it is a difficult challenge to generate a considerable reinforcing effect at a low filler concentration by melt mixing [42]. Moreover, GNPs are still more expensive than CB, hence CB with low cost is the most used industrial filler in the rubber industry [7]. CB-GnP hybrid fillers are a new research area for the development of the EPDM-based composites with desired properties at relatively lower filler loading and to solve the agglomeration problem caused by graphene.

To date, several research teams have prepared and studied the properties of EPDM/GnP composites [4,6,17,18,19,22,43], while, to the best of our knowledge, there is no reference available that interpreted the size effect of GnPs on the EPDM composites. Besides this, our aim is also to develop thermally stable rubbery materials suitable to be applied in the future for hydrogen energy transport, i.e., with low gas permeability. In this study, firstly the size effect of GnPs on the mechanical, viscoelastic, swelling, and gas barrier properties of EPDM-based composites are investigated to determine the optimum reinforcement performance of GnPs. Then, GnPs exhibiting the best performance are used as second filler materials for the EPDM/CB composites prepared by melt mixing to investigate the effect on the above-mentioned properties of the hybrid composites. The 2D nanoparticles such as GnPs have a reinforcing effect on the mechanical properties of the elastomeric matrix. Finding synergistic effects of the presence of two or more different types of nanofillers in the elastomeric matrix in order to achieve a substantial increase in barrier properties of new materials suitable for selected applications mainly as sealing and separation elements with low gas permeability was also the aim of the study.

## 2. Materials and Methods

### 2.1. Materials

EPDM, Keltan 4450S, including 52 wt.% ethylene monomers with the viscosity of 42 MU (ML (4 + 1), 125 °C) and a specific density of 0.86 kg/dm^3^ was supplied by SpecialChem (New York, NY, USA). Four kinds of GnPs were provided by Nanogarafi (Ankara, Turkey). The GnPs were labeled G1, G2, G3, and G4, and their physical properties are provided in Table 1. Carbon black (N 550) was supplied by MAKROchem S.A. (Lublin, Poland). All ingredients were used as supplied without any further functionalization or purification process. All used ingredients containing matrix, fillers, and others (oil, 2,2,4-trimethy1-1,2-dihydroquinolin (AOX-TMQ), zinc oxide (ZnO), stearic acid, tetra-methyl thiuram disulphide (TMTD), and sulphur are listed in Table 2.

### 2.2. Preparation of Nanocomposites

The samples were prepared by melt mixing in a Brabender Plasticorder PLE 331 internal mixer (Brabender GmbH, Duisburg, Germany) with a 30 mL mixing chamber at 100 °C for 7 min of total mixing time at 50 rpm of mixing speed. All ingredients were fed to the mixer at a mixing speed of 30 rpm. The compound recipe for samples and process times are provided in Table 2. Firstly, ethylene–propylene–diene monomer (EPDM) was melt mixed for 1 min, then, nanofillers, AOX-TMQ, ZnO, and stearic acid were mixed prior to being added to the Brander mixer and mixed for 3 min, oil was added followed by continuous mixing for 2 min. For the composites with carbon black, half of the amount of carbon black was added in the second step, while the other half was added with the oil. Finally, TMTD and sulphur were fed to the mixer, respectively, and mixing was continued for 30 s after each addition. The sample undergoing the mixing process was further calendared using a laboratory two-roll mill (Nishimura, Tokyo, Japan). The calendaring process was conducted through sheeting, rolling, and sheeting steps at room temperature. This procedure was repeated four times with a rolling space of 5 mm, four times with a rolling space of 2 mm, and finally two times sheeting with a rolling space of 0.5 mm. The samples for the measurements were vulcanized using compression molding by using a hot press (Fontijne 200, Fontijne, The Netherlands) at 160 °C without pressure for 30 s and under pressure (5 MPa) for 10 min, then solidified by air cooling.

### 2.3. Characterization

To characterize the surface composition of GnPs and CB, X-ray photoelectron spectroscopy (XPS) measurements were performed on a Thermo Scientific K-Alpha XPS system (Thermo Fisher Scientific, Oxford, UK) with a monochromatic Al Kα X-ray source. The structure of GnPs and selected EPDM nanocomposites was observed using scanning electron microscopy (SEM, JEOL 5310-LV, Tokyo, Japan). EPDM nanocomposites were freeze-fractured in liquid nitrogen for SEM analysis. All samples were sputtered with gold before observation.

Dumbbell-shaped specimens with a working area of 30 × 4 × 1 mm were cut from the vulcanized sheet using a pneumatic toggle press (APK 4L, Tinius Olsen LTD, Redhill, UK). The tensile measurements of samples were carried out by using an Instron 3365 (Instron, High Wycombe, UK) universal testing machine with a cross-heat speed of 50 mm/min at room temperature. The mean values and standard deviations of six tests were reported for each sample. Dynamic mechanical analysis (DMA) measurements of EPDM-based nanocomposites were performed on a TA Instrument dynamic mechanical analyzer DMA Q800 (TA Instruments, Elstree, UK) in the temperature range, from −70 to 60 °C. The samples of uniform shapes were measured in the module tensile multi-frequency strain at 10 Hz with a strain amplitude of 5 μm and a heating rate of 2 °C/min.

Swelling measurements of vulcanized and non-vulcanized unfilled EPDM and EPDM-based nanocomposites were performed in toluene at room temperature. The dimension of the samples was approximately 4 × 30 × 1 mm^3^. Each sample was weighed using a highly sensitive analytical balance and dipped into 5 mL of toluene for 1 week. This time was determined using preliminary experiments as sufficient for reaching the equilibrium swelling degree, which was usually leveling off after less than 48 h. The swelling was measured every 2 h until samples reached the constant weight. The gravimetric method was used to determine the swelling degree:

(1) $$\xi =\frac{m-m_{0}}{m_{0}}$$

where *m*_0_ and *m* refer to the sample weight before swelling and the weight of the swollen sample, respectively. The crosslink density was determined using the Flory–Rehner equation:

(2) $$v_{e}=-\frac{ln\left(1-V_{r}\right)+V_{r}+\chi V_{r}^{2}}{V_{s}\left(\sqrt[{3}]{V_{r}}-0.5V_{r}\right)}$$

where the molar volume of solvent depicts *V_s_*, 106.3 cm^3^/mol for toluene, and *χ*, the Flory/Huggins interaction parameter between EPDM and toluene, taken as 0.496 [22]. The rubber volume fraction *V_r_* was determined using Equation (3):

(3) $$V_{r}=\frac{m_{0}}{m_{0}+\eta \left(m-m_{0}\right)}$$

*η* refers to the density ratio of elastomer to toluene.

According to ASTM D 1434, the gas permeability measurements of the EPDM-based composites were carried out with a VAC-V1 (Labthink GmbH, Neu-Isenburg, Germany) differential pressure method of the gas permeation apparatus, which is controlled by the OX2/230 OTR test system (Labthink GmbH, Neu-Isenburg, Germany).

## 3. Results and Discussion

Before starting composite preparation, surface analysis of all used fillers was completed with the aim of comparison of GnPs surface properties, oxidation state, and morphology investigation All obtained parameters can be helpful for an explanation of composite properties, especially when hybrid fillers are used.

### 3.1. X-ray Photoelectron Spectroscopy Analysis of Carbon-Based Materials

**Figure 1:**
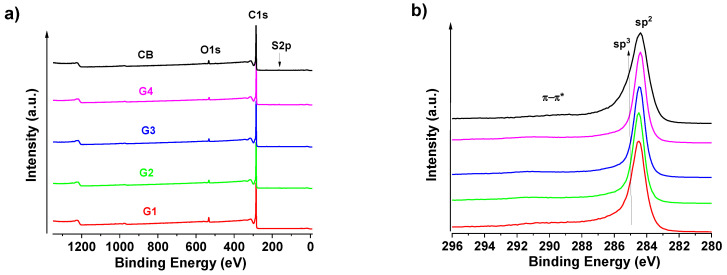
XPS comparison of the (**a**) survey and (**b**) C1s region of GNPs and CB.

Figure 1a presents the XPS survey of all carbon-based fillers in the range from 10 to 1350 eV. The XPS results of CB and GnPs are provided in Tables S1 and S2. As proposed, a large amount of carbon (C 1s at ca. ≈ 284.4 eV), but also oxygen (O 1s at ca. ≈ 531.4 eV) and a small amount of sulphur (S 2p at ca. ≈ 163.8 and 168.1 eV) were detected. The C1s at ≈284.4 eV with an asymmetry and symmetric loss peaks (labeled π−π*) is typical for a sp^2^ carbon atom (Avantage software 5.9929, XPS knowledge database (Thermo Fisher Scientific, Eastbourne, UK)). In Tables S1 and S2, the deconvolution fit for the signals of C 1s and O 1s was also performed. Besides graphitic carbon signals (sp^2^ at ca. 248.4 eV and sp^3^ at 284.7 eV), signals for C–O (ca. 286.4 eV), C=O (287.2 eV), and OC=O (289.2 eV) groups were detected (see also Figure 1). These signals correspond to the O 1s signals of C=O aromatic (C=O_ar_, centered at ca. 531.4 eV), OC=O_x_ (532.5 eV), C–O (ca. 533.5 eV), and O2C=O (ca. 535.7 eV) groups. The signals of C 1s as well as their apparent surface chemical compositions for all GnPs are almost the same. Among all types of GnPs, the G4 indicate the lowest atomic content of C 1s (95.6%) and its surface is the highest oxidized one. Additionally, this increased oxidization degree of G4 is easily shown by a higher amount of carbonyl (C=O_ar_) in the case of the O 1s signal. In all cases, there is a small amount of sulphur (S 2p at ≈164–168 eV) coming probably from the production method of GNP preparation. CB has clearly the highest portion of sp^3^ carbon indicating the presence of amorphous carbon, this is also visible from Figure 1b and the broader C1s peak for CB.

### 3.2. Morphology Analysis

#### 3.2.1. Morphology Analysis of Materials

**Figure 2:**
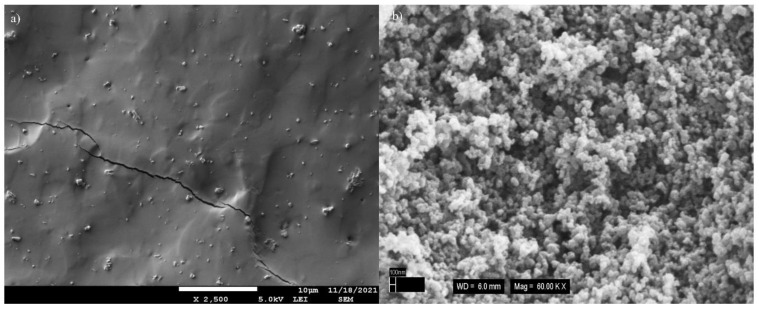
SEM images of (**a**) unfilled EPDM and (**b**) CB.

**Figure 3:**
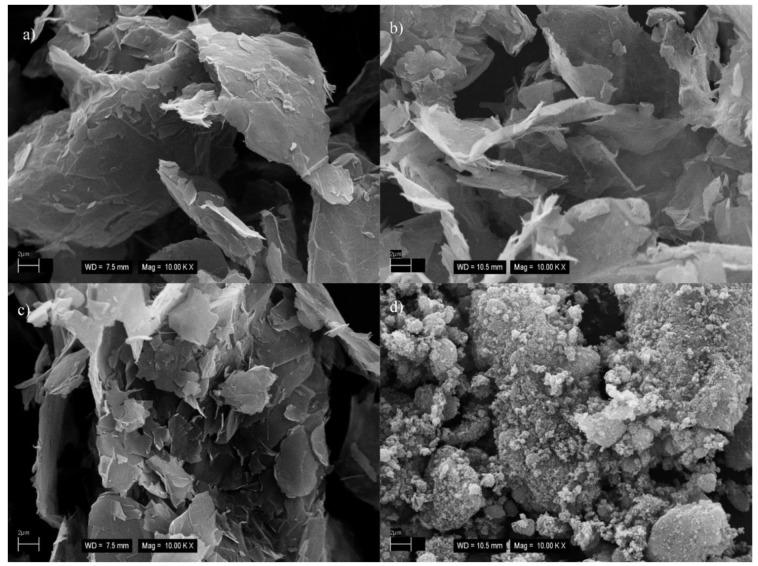
SEM images of (**a**) G1, (**b**) G2, (**c**) G3, and (**d**) G4.

For detection of the composite structure, SEM micrographs of vulcanized cryo-fractured EPDM prepared using melt mixing with all stabilizers and crosslinking agents were studied firstly. Carbon black forms aggregates composed of primary particles combined into units that cannot be easily separated. We studied the morphology of the CB N 550 used for composite preparation. SEM images of the unfilled EPDM and CB are provided in Figure 2a,b, respectively. As seen in Figure 2a, the fracture surface of unfilled EPDM is smooth and flat with typical brittle fracture patterns. Additionally, the SEM image of unfilled EPDM shows ZnO particles. In Figure 2b, CB particles form aggregates and CB cannot be observed as a single particle. We used four different GnPs particles, therefore, the morphology investigation was undertaken primarily to check the structure and particle size of these fillers. All GnPs structures are depicted in Figure 3a–d, respectively. In Figure 3, aggregated forms of GnPs were observed due to the high surface energy of GnPs. Figure 3a–c show that all types of GnPs are irregularly shaped plates of G1, G2, and G3 with many sharp edges. Figure 3a presents clearly that G1 has the largest lateral size compared to others. As seen in Figure 3d, the G4 structure differs from the others, indicating high agglomeration caused by higher surface energy due to its substantially higher specific surface area.

#### 3.2.2. Morphology Analysis of EPDM/GnPs Composites

Firstly, EPDM composites with GnPs filler were prepared, containing up to 20 phr of four various types of GnPs, labeled as G1, G2, G3, and G4. While G1, G2, and G3 have the same specific surface area and thickness, but different diameters. G4 has the highest values of specific surface area, lowest thickness, and diameter among all four nanofillers (Table 1). Then, the influence of the synergic effect of GnPs and CB on these properties is evaluated. G1 provides the optimum value in all features, G1 is selected as the second filler for the EPDM/CB/GNPs composites. Moreover, keeping the total filler consideration at a reasonable level is the most significant consideration to overcome the fabrication difficulties and keep the final composites economical, GnPs loading was varied up to 7 phr to investigate the synergy effect of CB and GnPs. Based on the studies on EPDM/CB composites, the variation in CB is 20 and 50 phr due to the percolation threshold of these composites. We investigated the morphological, mechanical, DMA, swelling, and oxygen permeability properties of EPDM-based composites with respect to GnPs.

**Figure 4:**
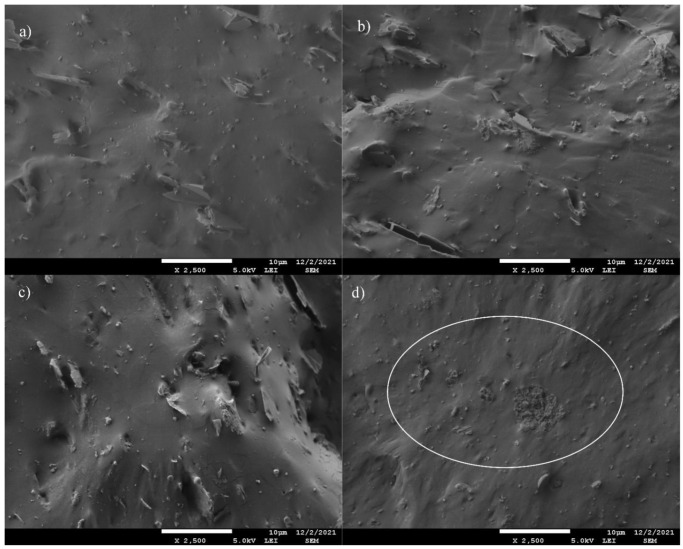
SEM images of (**a**) EPDM/G1, (**b**) EPDM/G2, (**c**) EPDM/G3, and (**d**) EPDM/G4 composites with 7 phr of GnPs (the white ellipsis shows the agglomeration of G4 into the EPDM matrix).

SEM micrographs of four types of EPDM/GnPs composites with 7 phr of GnPs are depicted in Figure 4a–d. As mentioned by Dash et al. [8], the filler dispersion is a significant parameter to attribute improved features of EPDM-based composites. It is observed that GnPs uniformly and randomly disperse in the EPDM matrix. The uniform distribution of fillers into EPDM leads to better load transfer between the filler and matrix. The better mechanical properties can be attributed to it. It is seen that GnPs projected out from EPDM and GnPs are covered by EPDM layers, which also indicates good filler adhesion to the polymeric matrix. Two various dispersions of GnPs in the EPDM matrix are observed: aggregated and separately dispersed. While G1, G2, and G3 indicate separately dispersed in EPDM, the aggregation takes place in the EPDM/G4 composite as shown in Figure 4d. As a result of this agglomeration, more continuous pathways formed because of close contact of G4 particles compared to others [8]. The reason may be attributed to the small dimension and higher surface energy of G4. In addition, some voids are observed in the EPDM/GnPs composites with G2 and G3.

#### 3.2.3. Morphology Analysis of Hybrid Composites

**Figure 5:**
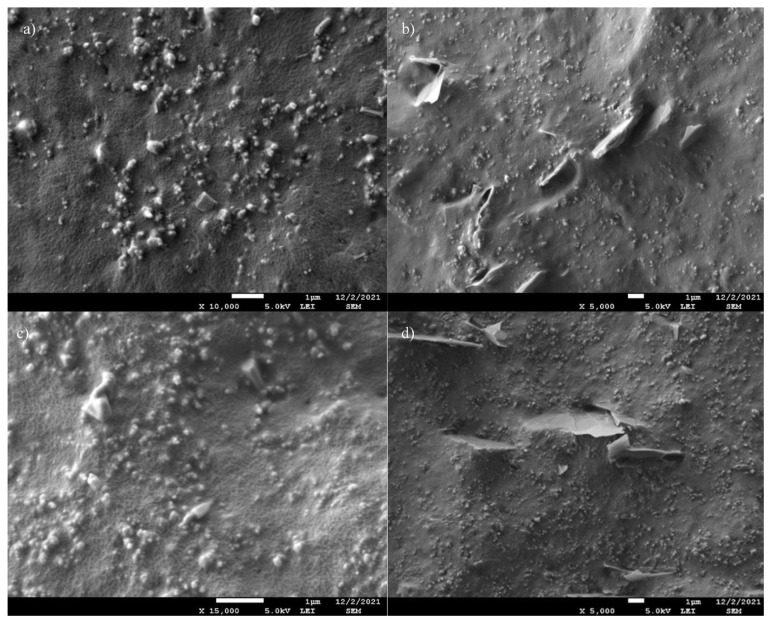
SEM images of (**a**) EPDM/CB20, (**b**) EPDM/CB20/G1_7phr, (**c**) EPDM/CB50, and (**d**) EPDM/CB50/G1_7phr nanocomposites.

Figure 5a–d indicates the SEM micrographs of EPDM/CB/GnPs composites. While the unfilled EPDM shows a smooth surface (Figure 2a), the EPDM hybrid composites demonstrate a more fluctuant and rougher fracture surface including a deeper tear morphology, similarly observed in Lei et al. [6]. In Figure 5a–d, it can be seen that GnP layers are projected out from the EPDM matrix. CB particles indicate poor distribution through EPDM with apparent aggregates of CB [7]. It is clearly seen in Figure 5c that CB agglomerates are connected and form a network throughout the EPDM matrix. As seen in Figure 5a, CB can create a network in the EPDM-based nanocomposites with 20 phr of CB, however, CB is not continuously dispersed, and filler-rich or EPDM-rich regions can be observed. In the case of the EPDM-hybrid composite with 20 phr of CB (Figure 5b), small gaps are detected which results in poorer adhesion and compatibility between the EPDM matrix and CB or GnPs.

### 3.3. Mechanical Testing Results

The enhancement in the tensile properties of elastomer composites is based on several parameters, such as the interfacial interaction between the matrix and filler, the interactions between the filler and other components, e.g., activators and accelerators, and filler dispersion in the matrix [1]. Generally, it is believed that the mechanical properties can enhance when efficient stress transfer at the filler/polymer interface and good filler dispersion are guaranteed [6].

**Figure 6:**
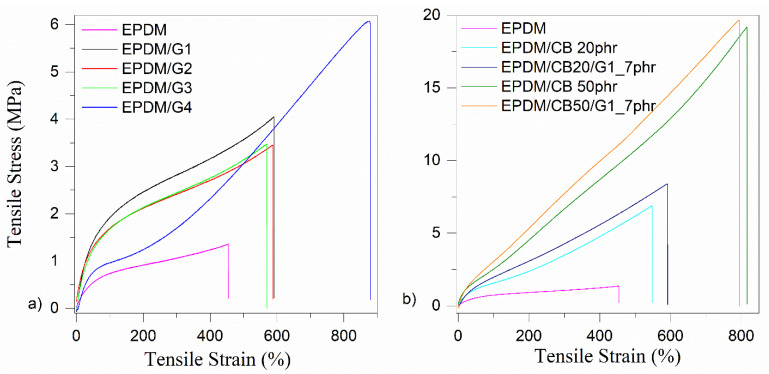
Stress–strain graph of (**a**) unfilled EPDM and EPDM/GnPs nanocomposites with 20 phr of fillers and (**b**) hybrid nanocomposites with 7 phr of G1 and various amounts of CB.

The stress–strain curves of EPDM/GnPs composites with 20 phr and EPDM-based hybrid composites with 7 phr of G1 are presented in Figure 6a,b, respectively. All stress–strain curves have an S shape, typical for vulcanized rubbers filled with reinforcing filler. In the case of hybrid nanocomposites, the stress–strain relationship is almost linear within the whole range. All vulcanizates exhibit the inflex point on the stress–strain curve, which appears for all mixtures at a deformation of slightly above 200%. An important observation consists of the fact that while for EPDM filled with G1, G2, and G3, the break occurs soon after the inflex point on the stress–strain curves, EPDM filled with G4 exhibits a quite extensive deformation also above the inflex point. This part is ascribed to the orientation of rubber chains, forming structures of polymeric chains ordered in the direction of the drawing. This orientation is even supported to a certain extent by lower crosslink density so that larger rubbery segments can be ordered contributing to a significant increase in the tensile strain accompanied by the increase in the strain at break.

#### 3.3.1. Mechanical Testing Results of EPDM/GnPs Composites

The values from the testing of mechanical properties (Young’s modulus, strain at break, stress at tensile strength, M100, and M300) on GnP loadings for EPDM-based composites are provided in Table S3. As expected, except for the samples reinforced with G4, the Young’s modulus of EPDM/GnPs composites increases linearly with increasing GnPs loading compared to the unfilled EPDM. This increase can be attributed to the higher Young’s modulus, good dispersion, and high aspect ratio of GnPs, and good interfacial adhesion of the filler and matrix. The inclusion of the filler into the matrix can restrict the mobility of polymer chains and increase the crosslink ratio [44]. Within the experimental range of filler loading, the EPDM-based composites including G4 exhibit lower Young’s modulus values than the unfilled EPDM. This can be attributed to the agglomeration form of G4 (Figure 3d) and the worse dispersion of G4 (Figure 4d) into the EPDM matrix. The agglomeration can lead to defects in the composites.

The Young’s modulus of composites increases from 1.61 MPa for the unfilled EPDM to 4.12, 3.53, and 3.73 MPa for the EPDM/G1, EPDM/G2, and EPDM/G3 composites, respectively, and decreases to 0.87 MPa for the EPDM/G4 composites when GnPs content increases from 0 to 20 phr. An increase in Young’s modulus is more noticeable for the EPDM/G1 composites compared to others. This may be due to better dispersion of G1 in the EPDM matrix. When compared in terms of specific surface area, GnPs with lower specific surface area (170 m^2^/g) give better results than ones with higher (800 m^2^/g). The reason is the agglomeration of filler with a higher surface area due to higher surface energy because of its smaller dimension. Based on the results, for a fixed specific surface area, Young’s modulus is dependent on the lateral size of GNPs, GnPs with a larger lateral size achieve a higher value of Young’s modulus above 7 phr of GnPs. Up to this concentration, there is no appreciable difference in the values of Young’s modulus among the EPDM/G1, EPDM/G2, and EPDM/G3 composites.

**Figure 7:**
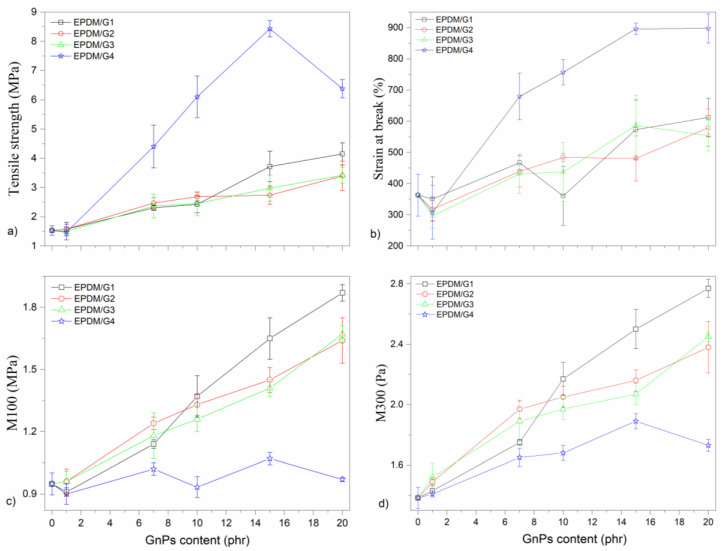
Mechanical properties of EPDM filled with various concentrations of different GnPs: (**a**) tensile strength, (**b**) strain at break, (**c**) M100, and (**d**) M300.

The values of mechanical properties, namely tensile stress, strain at break, as well as moduli M100 and M300 for vulcanizates of EPDM filled with various concentrations of each GnPs are shown in Figure 7. It is seen that while the concentration dependencies for G1, G2, and G3 are similar or even for some parameters almost identical (e.g., strain at break, Figure 7b), the dependencies for the G4 substantially differ. Both tensile stress and strain at break are close to double values compared to the three other GnPs, while the values for moduli M100 and M300 are significantly lower for EPDM filled with G4. Surprisingly, the increase in both moduli with rising G4 concentration is almost negligible, unlike the courses for the other three kinds of GnPs.

Figure 7a shows that the tensile strength of the composites with all kinds of GnPs increases with increasing GnPs contents. An unfilled EPDM includes crosslinked molecular chains being kinked, twisted, and coiled. These molecular chains partly straighten, uncoil, and untwist, which leads to elongation in the uniaxial stress direction. The rupture of these chains happens as the loading passes over a critical value, resulting in the strain at fracture. The inclusion of GnPs into the EPDM matrix forms three effects: 1—providing strength and stiffness due to stiffer and stronger GnPs; 2—forming more crosslink points of physical nature to crosslinked molecular chains formed by sulphur during a chemical reaction (vulcanization); and 3—behaving as a connector bridging various EPDM chains, causing an increase in strain at break. Enhancing the strength and stiffness with the addition of GnPs is explained by two former effects. With the increase in GnP loading, the increase in tensile strength is observed because GnPs strengthen and stiffen the EPDM matrix [22].

At 20 phr of GnPs loading, the enhancements of tensile strength of EPDM-based composites including G1, G2, G3, and G4 were ~171%, 122%, 123%, and 318%, respectively. The EPDM/G4 composites show the highest tensile strength compared to others at different GnP loadings. The tensile strength reaches the maximum at 15 phr of G4 which is 8.43 MPa. Since G4 has the highest specific surface area and the smallest dimension, it exhibits higher surface activity, resulting in better strengthening and stronger adsorption [5]. At 20 phr of G4, there is an abrupt decrease in the tensile strength of EPDM/G4 composites, but still its value is higher than that of the others at the same concentration. Because of this, the agglomeration form of G4 leads to defects at high concentrations. For types of GnPs with the same thickness (5 nm) and specific surface area (170 m^2^/g), the optimum lateral size is approximately 30 µm over 18 and 7 µm above 10 phr of GnPs. Up to this concentration, it is observed that the gradual addition of GnPs into the EPDM matrix progressively increases the tensile strength, however, the values of the EPDM composites with G1, G2, and G3 are more or less the same. Moreover, at all concentrations of the filler loading, G2 and G3 give a nearly identical mechanical performance. Consequently, these results indicate that the specific surface area is a more significant parameter for tensile strength than lateral size.

For all series of EPDM/GnPs composites, regardless of GnPs’ dimensions, while at 1 phr of GnPs, the elongation at break drops down, there is a considerable enhancement in the elongation at break with progressive inclusion of GnPs (Figure 7b). This can be attributed to C=C on the surface of GnPs, which can participate in the curing process and create additional crosslinks to resist the outside force. Additionally, upon the outside force action, GnPs can be exfoliated. This may be the reason for the initial decrease in elongation at break [43]. On a comparative basis, the enhancement in elongation at break is found to be ~69%, ~60%, ~53%, and ~147% (from ~363% for the unfilled EPDM to ~612%, ~579%, ~553%, and ~897%) for the EPDM/G1, EPDM/G2, EPDM/G3, and EPDM/G4 composites, respectively. Compared to types of GnPs, it is obvious that the elongation at break exhibits a similar behavior as the tensile strength. The composites with a higher specific surface area have a higher mechanical performance. 

Upon loading, GnPs can efficiently share a large stress portion and act as physical crosslinks increasing elastomer density and the total crosslink density. This causes a high strain at break [21]. The reason for this behavior is almost entirely high specific surface area.

M100 and M300 of unfilled EPDM are 0.95 and 1.38 MPa and are summarized in Table S3. As seen in Figure 7c,d, excluding the composites with G4, M100 and M300 increase with the increase in the GnPs loading in the system. Comparing M100 and M300 of the EPDM/GnPs composites at a certain GnPs loading, the highest value was observed in the 20 phr G1 filled system. However, as compared to G2 and G3 reinforced composites, they are close to each other. As compared to GnPs with the same thickness (5 nm) and specific surface area (170 m^2^/g), the optimum lateral size is almost 30 µm over 18 and 7 µm above 10 phr of GnPs. The surface area of GnPs influences M100 and M300. The lower the surface area, the higher values of M100 and M300.

The data for M100 and M300 support the certain correlation of moduli with crosslink density [16], even for M100 this is clearly demonstrated since the material filled with G1 exhibits the highest crosslink concentration, EPDM filled with the G2 and G3 shows almost the same crosslink density, being somewhat lower compared to G1 and the crosslink density of EPDM filled with G4 is the lowest, corresponding with both M100 and M300. The reason for the effect of GnPs on the crosslink density can be possibly seen in the physical interactions of the graphene surface deactivating to a certain extent the activity of the crosslinking system, either the accelerators or perhaps also sulphur. In both cases, the final crosslink density would decrease, as was observed as seen in Table 5 with the crosslink densities data. The data for the M100 and M300 are supported also by values of storage moduli presented in the supplementary data in Figure S1.

Consequently, all types of EPDM/GnPs composites exhibit significantly higher mechanical parameters than the unfilled EPDM, a similar observation is made by Lu et al. [19]. The enhanced mechanical properties can be attributed to the superior mechanical properties of GnPs (tensile strength of 130 GPa and a capability to elongation one-fourth of the original length of GnPs during loading). In addition, there may be an adequate interfacial interaction between matrix and fillers because of good GnPs dispersion in the EPDM matrix, this can enhance the mechanical property through the stress transfer phenomenon [19]. It can be observed that the size and specific surface area of GnPs have a significant effect on the mechanical properties of EPDM-based composites. While G1 shows better enhancement in Young’s modulus, M100, and M300, G4 gives better results in other properties.

Obviously, the size of GnPs is a less important key factor than the surface area of GnPs, and the ruling parameter for tensile strength as well as the strain at break seems to be the surface area, which is the nearly same for G1, G2, and G3 but substantially larger for G4. On the other hand, during mechanical deformation a certain extent of additional exfoliation may occur which leads to the increase in the surface area available for the interactions with the matrix resulting in the increase in the content of physical crosslinks.

#### 3.3.2. Mechanical Testing Results of Hybrid Composites

**Figure 8:**
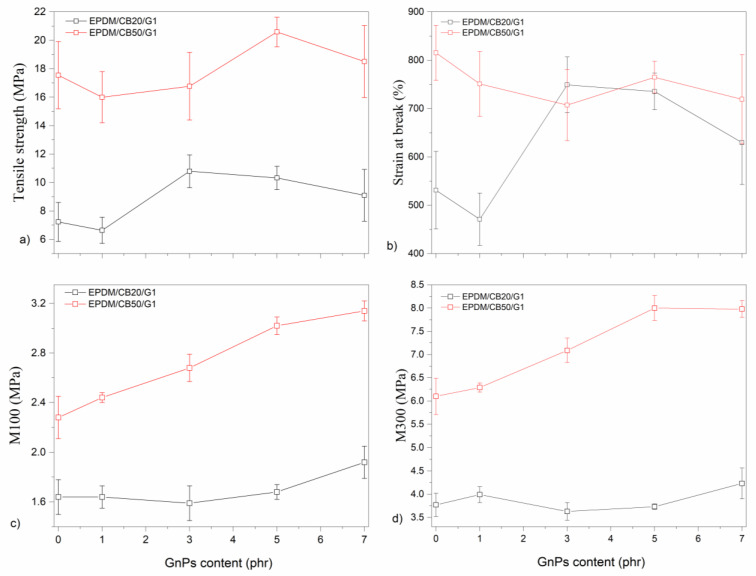
Mechanical properties of hybrid composites: (**a**) tensile strength, (**b**) strain at break, (**c**) M100, and (**d**) M300.

Figure 8 demonstrates tensile stress, and strain at break as well as moduli M100 and M300 for the hybrid composites. The values from testing of mechanical properties (Young’s modulus, strain at break, stress at tensile strength, M100, and M300) on GnPs loadings for hybrid composites are provided in Table S4. Composites with CB at 20 phr exhibit an increase in Young’s modulus, tensile strength, and elongation at break, respectively, by ~11%, ~373% ~46% in comparison with the enhancements ~79%, ~1047%, and ~125% made by filling with CB at 50 phr. These enhancements are attributed to intense specific interactions with the matrix. Additionally, the progressive inclusion of CB leads to a reduction in the distance between CB particles. The transition point of tensile strength as a function of CB content just expresses a critical distance between particle and particle, therefore, the excellent strengthening effect begins. A critical particle–particle distance is considered to be an essential prerequisite for achieving a highly efficient strengthening [5].

All mechanical properties oscillate and fluctuate with the addition of GnPs into the EPDM/G1 composites. In other words, there is no linear relation between Young’s modulus and GnPs loadings into EPDM/CB composites. The Young’s modulus of the EPDM composites including 50 phr of CB and 5 phr of GnPs is 3.18 MPa, being the highest and 97.5% higher than that of the unfilled EPDM. Excepting 7 phr of GnPs, all hybrid composites including 50 phr of CB show more or less the same level as one with only 50 phr of CB. Similar behavior can be observed in the tensile strength. The addition of 5 phr of GnPs enhances the tensile strength up to ~17% compared to one with 50 phr of CB. The elongation at break decreases with the addition of GnPs into EPDM/CB composites with 50 phr due to the agglomeration of filler, leading to voids in the composites.

In the case of the EPDM hybrid composites with 20 phr of CB, the improvement in tensile properties can be due to a better reinforcement effect of GnPs on EPDM as compared with single CB filler [45] and homogenous dispersion of CB-GnPs in the EPDM matrix [6]. In this sense, the improvements in the mechanical properties can also verify the uniform dispersion of GN-CB in the EPDM networks [6].

The improvement in the stiffness of composites can be attributed to the synergic effect of fillers, as described by Cai et al. [46]. The enhanced tensile strength of hybrid composites may be attributed to the good dispersion of CB and GnPs through the EPDM-based composites. Small particle sizes of CB can provide a large specific surface area and interact with the matrix, and this can allow more effective stress transfer [1]. The considerable enhancement in mechanical properties of EPDM/CB composites by the inclusion of GnPs can be attributed to superior mechanical properties of GnPs as well as great interfacial interaction and good dispersion of CB and GnPs [6].

In the case of hybrid composites with 50 phr of CB, the addition of G1 into the EPDM/CB composites leads to an increase in M100 and M300. These values are slightly influenced by the incorporation of G1 into ones with 20 phr of CB. It can be observed that there is a synergic effect of CB and GnPs on the M100 and M300 for the hybrid composites with 50 phr of CB, in the case of the ones with 20 phr of CB, any synergic effect is not observed.

Briefly, the concentration of the total fillers and each filler are important to achieve considerable improvement in mechanical properties due to the synergic effect of fillers.

### 3.4. Dynamic Mechanical Analysis

#### 3.4.1. Dynamic Mechanical Analysis of EPDM/GnPs Composites

**Figure 9:**
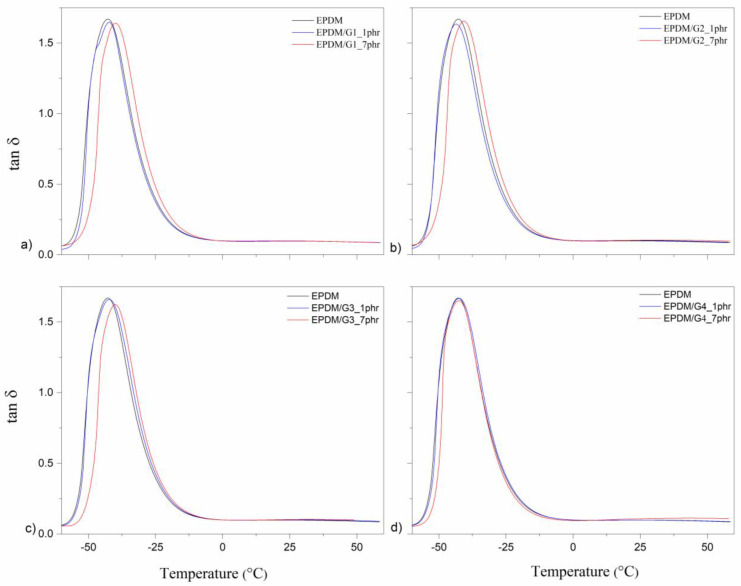
Tan δ for (**a**) EPDM/G1, (**b**) EPDM/G2, (**c**) EPDM/G3, and (**d**) EPDM/G4, composites.

Concerning the DMA results, only data for tan δ from unfilled EPDM and EPDM filled with two concentrations, 1 and 7 phr, of all GnPs are presented in Figure 9. It is seen that the curves are almost identical, concerning both the maximum temperature values as well as the height of the peaks. It means that interactions of GnPs surfaces with rubber matrix are not extremely high, although the interactions are not negligible. The height of the tan δ peak presents a slight increase with increasing GnPs loading and the position of the peaks slightly shifts toward a higher temperature. T_g_ refers to the maximum value of the tan δ curve [47].

As seen in Table 3, there is a small increase in the glass transition temperature as a function of GnPs loading. The height of the T_g_ exhibits a slight change with types of GnPs, a similar observation was described for elastomer composites [47]. This indicates that the presence of GnPs leads to a certain increase in the crosslinking density. A small shift to the higher maximum temperature, corresponding to T_g_ of the vulcanizate, is observed for G1 and G3, some shift may be seen also for G2 at 7 phr of the filler, but the EPDM filled with G4 does not exhibit any change in T_g_ compared to unfilled EPDM. Within the experimental range of specific surface area, the smaller the specific surface area of GnPs, the higher T_g_. In the other words, the T_g_ of unfilled EPDM and composites including the GnPs with a larger specific surface area (800 m^2^/g) is more or less the same for a fixed surface area (170 m^2^/g), and the lateral size of GnPs has a negligible effect on T_g_. It is also obvious that the area under the tan δ curves is in fact the same, indicating that the presence of GnPs does not affect the mobility of the rubber chains.

Additional DMA data, showing the temperature dependences of storage and loss moduli are presented in Figures S1 and S2, supporting the data presented above as well as mentioned in the next section on mechanical properties. At −60 to −40 °C, an abrupt decrease is observed in the storage modulus of samples. The storage modulus of composites increases with the increase in the content of GnPs at the experimental range of temperature. While this increase is more prominent at low temperatures (glass regions), the increase in the rubbery region is more moderate. The reason for the increase in the storage modulus is due to: (a) GnPs’ resilient nature because GnPs have 25% of fracture strain, a fracture strength of 100 GPa, and a modulus of 1 TPa, additionally, under loading, GnPs move the stress with sharing load fractions and restrict the EPDM molecules’ movement in the vicinity of GnPs; and (b) the presence of GnPs in the EPDM matrix behaving as additional physical crosslinking [22]. The pure EPDM indicates a storage modulus of 2.42 MPa at 10 °C, while a ~62%, ~57%, ~60%, and ~31% increase in the respective value is obtained by adding 7 phr of GnPs for EPDM composites with G1, G2, G3, and G4, respectively.

Figure S2, loss modulus vs. temperature (DMA damping), indicates that the motion of the crosslinked macromolecules of the EPDM matrix can dissipate and absorb fracture energy at various temperatures. Generally, the susceptibility of composites to tearing decreases with the increase in the area under the graph. The increase in the loss modulus is observed below the transition temperature. This implies a high damping property of samples, especially at low temperatures. This importantly highlights enhanced resistance to tearing added by GnPs [22]. For all series of composites, regardless of the specific surface area and lateral size of the GnPs, the loss modulus of composites enhances with increasing content of GnPs, specifically at low temperatures. At 10 °C, loss modulus of the unfilled EPDM is 0.236 MPa, while that of the EPDM composites with G1, G2, G3, and G4 is 0.370, 0.374, 0.379, and 0.312 MPa, increased by 57%, 58%, 61%, and 32%, respectively. The loss modulus indicates almost the same level of improvement as the storage modulus. It shows the same behavior in terms of the size effect of GnPs. 

#### 3.4.2. Dynamic Mechanical Analysis of Hybrid Composites

**Figure 10:**
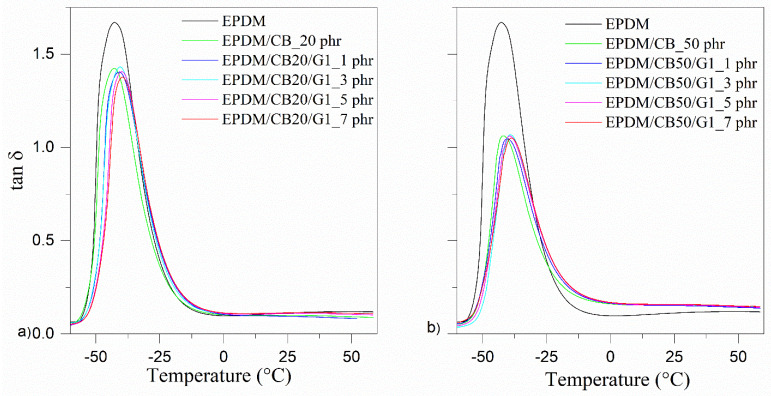
Tan δ for (**a**) EPDM/CB20/G1 and (**b**) EPDM/CB50/G1 composites.

The data for tan δ of unfilled EPDM and hybrid composites as a function of GnPs loading are presented in Figure 10 and Table 4. The height of the T_g_ peak exhibits a considerable decrease in the EPDM reinforced with 20 and 50 phr of GnPs. The T_g_ shifts moderately to a higher temperature when the content of GnPs increases from 1 to 7 phr. As mentioned above, the presence of GnPs has a slight effect on the mobility of rubber chains. When comparing two hybrid composites with the same GnPs loading, there is an inconsequential difference between them. Consequently, an identical synergic effect between CB and GnPs is not observed.

The influence of GnPs and CB can be observed from the storage modulus and loss modulus for the EPDM/CB/G1 composites as shown in Figures S3 and S4, respectively. At 10 °C, in the case of the composites with only CB, the storage modulus of the composites with 50 phr of CB is 13.85 MPa, while that of one with 20 phr of CB is 4.55 MPa, corresponding to 472% and 88% improvements, respectively. In the case of hybrid composites with 20 phr of CB, the storage modulus progressively increases with GnPs loading, it presents fluctuation for ones with 50 phr of CB. Mondal and Khastgir [48] claimed that the higher CB content helps with the delamination of GnPs layers which provides some enhancement in the filler–matrix interaction [48]. The storage modulus of hybrid composites is somewhat higher than that of EPDM/CB composites. GnPs in 2D layer form can create an inlaid network pathway with an EPDM matrix, which obtains a clear reinforcement effect. The interface properties of GnPs can influence the value of the storage modulus. The loss factor of composites increases with the increase in the filler content. The interface interaction between the matrix and fillers and dispersion of fillers in the matrix are enhanced, resulting in a more effective transfer in the interface stress.

### 3.5. Crosslink Density

#### 3.5.1. Crosslink Density of EPDM/GnPs Composites

The crosslink density of the EPDM-based composite is determined using the swelling method. The swelling ratio (ξ) and corresponding crosslinking density (υ_e_) values of EPDM/GnPs are provided in Table 5. The swelling ratio and crosslinking density of unfilled EPDM are 2.65 and 168.20 mol·m^−3^, respectively. In general, the inclusion of GnPs leads to a significant increase in the crosslinking density. There is an observation of 12.5%, 5.7%, and 4.9% decrease and 6% increase in the swelling ratio of EPDM-based composites with G1, G2, G3, and G4, respectively. The crosslinking density enhances by 33.9%, 13.1%, and 11.9% and reduces by 12.5% for G1, G2, G3, and G4, respectively. During melt mixing, the polymer macromolecules entangle with GnPs and they may move into the space between individual layers of GnPs. When keeping the same ratio of curing chemical to polymer in the preparation process, the presence of GnPs contributes to the formation of the physical crosslinking points, greatly enhancing the crosslink density [22]. As mentioned in the mechanical properties part, the physical interactions of the GnPs surface can hinder to a specific extent the activity of the crosslinking system, either the accelerators or maybe sulphur. The EPDM composites reinforced G4 exhibit a higher swelling ratio and lower crosslinking density than the unfilled EPDM. Moreover, the active surface area to crosslink with the EPDM molecules can decrease due to the aggregated form of G4. On a comparative basis, for a fixed specific surface area of GnPs (170 m^2^/g), the optimum lateral size for swelling ratio and crosslinking density is nearly 30 µm over 18 and 7 µm, G2 and G3 exhibit similar behavior.

Considering the data on crosslinking density and comparing them with mechanical properties presented in the previous section, a query may occur concerning the presence of G4 as the reinforcing filler. The reason may consist of the methods of determination of the corresponding parameters. If discussing tensile strength and elongation at break, the most important is the specific surface area, which is in fact ruling both parameters. Therefore, the addition of G4 is highly beneficial in achieving the highest strength parameters, apparently due to strong interactions between the GnPs surface and the EPDM matrix. In this case, the mechanical analysis was undertaken in the time period of a few minutes. On the other hand, the determination of crosslink density is calculated from data on equilibrium swelling, where the equilibrium is reached after 24 h or more. During this time, part of the physical crosslinks between G4 and EPDM may be destructed and are no more active in keeping the crosslinked structure together. It is even possible that the layered structure of GnPs is not completely exfoliated during melt mixing and may be maintained in the rubber composite. This is stable enough to resist the short mechanical load but may be destroyed by the less aggressive but the permanent effect of the solvent penetrating slowly between the layers and not only destroying them but being deposited between the GnPs layers increasing the measured solvent content.

#### 3.5.2. Crosslink Density of Hybrid Composites

The swelling ratio and crosslinking density of EPDM/CB/GnPs composites are provided in Table 6. As mentioned above, the presence of fillers in the EPDM-based composites leads to a decrease in swelling ratio indicating an increase in crosslinking density, except for G4. The increase and decrease show a fluctuation with increasing GnPs loading. While the decrease in swelling ratio is found to be ~36% (from 1.98 for 20 phr of CB to 1.46 for 50 phr of CB), the increase in the crosslinking density is ~86% (from 317.21 mol·m^−3^ for 20 phr of CB to 591.05 mol·m^−3^ for 50 phr of CB). With increasing CB loading, the abundant crosslinking network is mostly from EPDM chains’ crosslinking and the creation of the bound EPDM/CB structure by chemical bonding and physical adsorption between the molecular chains of EPDM and CB [7]. GnPs create the crosslinks between the macromolecules of polymer used to determine the crosslinking density into polymer composite.

### 3.6. Oxygen Permeability

Table 7 shows the oxygen permeability of unfilled EPDM matrix and composite samples. It is seen that the addition of fillers results in higher resistance to oxygen permeability. This effect is understandable providing that gas molecules are unable to penetrate through the compact carbon layer regardless of CB or GnPs. Therefore, in the simplest interpretation, the presence of the fillers has the same effect as the decrease in the cross-section of the tested rubber specimen in the direction perpendicular to the direction of the gas penetration. Filler particles create an obstacle extending the route the gas molecule must pass. Quantitatively, the efficiency of the filler presence regarding the decrease in gas permeability depends on the area of the filler particles oriented perpendicularly to the gas penetration direction. This area increases with the amount of the filler content in the matrix and with the orientation of the particles if the filler particles are anisotropic.

It is seen that the addition of CB results in a significant decrease in oxygen permeation, moreover, as expected, the increase in CB concentration leads to a further decrease in oxygen permeability. Similarly, the addition of GnPs also results in the gas permeation resistance, but gas permeability achieved with 20 phr of CB is obtained with 7 phr of GnPs. Obviously, the higher efficiency of GnPs is reached due to the layered anisotropic structure of the GnPs particles which hinders gas transfer extending the path of gas diffusion [49] more efficiently, compared to more or less the spherical shape of primary CB particles.

It is also seen that the application of hybrid fillers CB and GnPs presents a synergistic effect represented by a further significant decrease in gas permeability.

However, the shape geometry effect, although probably the most important, may be completed due to a number of other factors such as a reduction in free volume, increased density, and tortuosity resulting in reducing the available area for diffusion and increasing the distance that a solute must pass across the film as following a tortuous path around the impermeable fillers [50]. From this point of view, the changes in crosslink density might be questioned. Regardless, in our view, crosslinking density in our case should not affect the gas permeability significantly. First, the real density of cross bonds is approximately one junction for 400–1000 carbons in the main rubber chain. Further on, the crosslink is formed usually by two or more sulfur atoms so that its length allows sufficient mobility of the crosslinked segments and almost unhindered diffusion of gas molecules. Therefore, any comparison of the oxygen penetration data and crosslinking density seems to have no significance. Moreover, each crosslink, besides exhibiting the reinforcing effect introduces a defect in the rubber macromolecules which may affect density as well as the natural ordering of the macromolecules. All of that may even increase the gas permeability through the crosslinked material.

## 4. Conclusions

In this study, firstly, the EPDM/GnPs composites were fabricated by using the melt mixing method up to the filler concentration of 20 phr, followed by compression molding. Three types of GnPs with the same specific surface area and thickness, but different lateral sizes and one type of GnPs with high surface area and the lowest size were used as filler material. The influence of GnPs size on morphological, mechanical, and viscoelastic properties, swelling ratio, crosslinking density, and oxygen permeability was investigated. Then, in order to determine the synergic effect of CB and GnPs on the above-mentioned properties, the various content (up to 7 phr) of GnPs with lower surface area and the highest lateral size were added into EPDM/CB composites with 20 and 50 phr. The main conclusions are as follows:

1. Based on SEM results, the GnPs with low specific surface area show separate dispersion through the EPDM matrix, the one with the high surface area exhibits aggregated dispersal in the polymer matrix because of its high surface energy and small dimension. The addition of GnPs into the EPDM/CB composites results in better dispersion of the filler as compared to the composites with only CB.
2. Concerning the reinforcing effect, the specific surface area is the ruling parameter, while the differences in the lateral size have only a marginal effect, if any. For GnPs with a specific surface area of 170 m^2^/g, the lateral size effect of GnPs does not have a pronounced impact on tan δ and Tg of the EPDM/GnPs composites. GnPs with 800 m^2^/g show almost the same value as the unfilled EPDM.
3. Usually, for all series of EPDM-based composites, the crosslink density increases with increasing filler loading due to the formation of physical crosslinks. Only the composites including GnPs with a higher surface area (800 m^2^/g) exhibit the opposite of this behavior. For a given specific surface area of GnPs, 170 m^2^/g, the optimum lateral size for the swelling ratio and crosslinking density is nearly 30 µm over 18 µm and 7 µm. In the case of hybrid composites, the optimum GnPs loading is needed to obtain the lowest swelling ratio and the highest crosslinking density.
4. The size of GnPs has no considerable influence on the oxygen permeability in EPDM/GnPs composites, but a synergistic effect of CB and GnPs presence in the EPDM matrix is observed. Materials based on vulcanized elastomers with specific 2D fillers with gas permeability decreasing have interesting application potential.

## Figures and Tables

**Table 1:** Properties of GnPs from product datasheets.

Properties	G1	G2	G3	G4
Purity (%)	99.9	99.9	99.9	99.9
Thickness (t) (nm)	5	5	5	3
Diameter (D) (μm)	30	18	7	1.5
Aspect ratio (D/t)	6000	3600	1400	500
Specific surface area (m^2^/g)	170	170	170	800

**Table 2:** Formulation of EPDM nanocomposites.

Material	Content (phr)	Mixing Time (min)	Supplier
EPDM ^a^	100	0–1	SpecialChem (New York, NY, USA)
GnPs ^b^	1, 3, 5, 7, 10, 15, 20	1–4	Nanogarafi (Ankara, Turkey)
CB ^c^	20, 50	1–4	MAKROchem S.A. (Lublin, Poland)
Oil	10	4–6	Unipetrol (Bratislava, Slovakia)
AOX-TMQ ^d^	1	1–4	Nord Chemie (Alfianello, Italy)
ZnO ^e^	5		Oddział Huta Oława (Oława, Poland)
Stearic acid	1		OLEOCHEM, a.s (Ústí nad Labem-Střekov, Czechia)
TMTD ^f^	2.5	6–6.5	RDC S.r.l. (Bareggio, Italy)
Sulphur	0.5	6.5–7	CHEMICAL PLANTS “Siarkopol” TARNOBRZEG Ltd. (Tarnobrzeg, Poland)

^a^ Ethylene–propylene–diene monomer, ^b^ graphene nanoplatelets, ^c^ carbon black, ^d^ the antioxidant 2,2,4-trimethy1-1,2-dihydroquinolin, ^e^ zinc oxide, ^f^ tetra-methyl thiuramdisuphide as the vulcanization accelerator.

**Table 3:** Results from the DMA investigation of EPDM/GnPs composites, T_g_ using the tan δ peak.

GnPs (Phr)	EPDM/G1	EPDM/G2	EPDM/G3	EPDM/G4
0	−42.68	−42.68	−42.68	−42.68
1	−42.16	−43.63	−42.18	−42.51
7	−39.88	−40.67	−39.99	−42.44

**Table 4:** Results from the DMA investigation of EPDM/CB/G1 composites, T_g_ using the tan δ peak.

G1 (Phr)	EPDM/CB20/G1	EPDM/CB50/G1
Unfilled EPDM	−42.68	−42.68
0	−41.89	−42.81
1	−40.24	−40.80
3	−39.14	−40.51
5	−39.39	−39.79
7	−38.70	−39.56

**Table 5:** Swelling ratio (ξ) and crosslinking density (υ_e_) of the EPDM/GnPs composites with 7 phr of filler.

Samples	ξ	υ_e_ (mol·m^−3^)
Unfilled EPDM	2.65	168.20
EPDM/G1	2.32	224.90
EPDM/G2	2.50	190.45
EPDM/G3	2.52	188.00
EPDM/G4	2.81	146.78

**Table 6:** Swelling ratio (ξ) and crosslinking density (υ_e_) of the EPDM-based hybrid composites.

G1 (Phr)	EPDM/CB20/G1		EPDM/CB50/G1	
	ξ	υ_e_ (mol·m^−3^)	ξ	υ_e_ (mol·m^−3^)
Unfilled EPDM	2.65	168.20	2.65	168.20
0	1.98	317.21	1.46	591.05
1	1.81	380.57	1.38	658.02
3	2.09	281.54	1.41	631.60
5	1.93	334.86	1.37	671.80
7	1.89	349.48	1.34	699.37

**Table 7:** Oxygen permeability of EPDM-based composites.

Sample	Oxygen Permeability (cm^3^/m^2^·d·atm)
Unfilled EPDM	1539
EPDM/G1 (7 phr)	1122
EPDM/G4 (7 phr)	1106
EPDM/CB (20 phr)	1244
EPDM/CB20/G1 (7 phr)	971
EPDM/CB (50 phr)	1063
EPDM/CB50/G1 (7 phr)	815

## Data Availability

The data presented in this study are available on request from the corresponding authors.

## Supplementary Materials

The following supporting information can be downloaded at: https://www.mdpi.com/article/10.3390/polym14194043/s1, Table S1: Apparent surface chemical composition of CB as determined using XPS; Table S2: Apparent surface chemical composition of GnPs as determined using XPS; Table S3. Mechanical properties of EPDM/GnPs composites; Table S4: Mechanical properties of EPDM/CB/G1 composites; Figure S1: Storage modulus (E′) for (a) EPDM/G1, (b) EPDM/G2, (c) EPDM/G3, and (d) EPDM/G4, composites; Figure S2: Loss modulus for (a) EPDM/G1, (b) EPDM/G2, (c) EPDM/G3, and (d) EPDM/G4, composites; Figure S3: Storage modulus (E′) for (a) EPDM/CB20/G1 and EPDM/CB50/G1 composites; Figure S4. Loss modulus for (a) EPDM/CB20/G1 and EPDM/CB50/G1 composites.

## References

[1] Mohd Zaini N.A., Ismail H., Rusli A. (2018). Sepiolite Hybridized Commercial Fillers, and Their Effects on Curing Process, Mechanical Properties, Thermal Stability, and Flammability of Ethylene Propylene Diene Monomer Rubber Composites. Iran. Polym. J. (Engl. Ed.).

[2] Valentini L., Bittolo Bon S., Lopez-Manchado M.A., Verdejo R., Pappalardo L., Bolognini A., Alvino A., Borsini S., Berardo A., Pugno N.M. (2016). Synergistic Effect of Graphene Nanoplatelets and Carbon Black in Multifunctional EPDM Nanocomposites. Compos. Sci. Technol..

[3] Mensah B., Kim S., Arepalli S., Nah C. (2014). A Study of Graphene Oxide-Reinforced Rubber Nanocomposite. J. Appl. Polym. Sci..

[4] Araby S., Su X., Meng Q., Kuan H.C., Wang C.H., Mouritz A., Maged A., Ma J. (2019). Graphene Platelets versus Phosphorus Compounds for Elastomeric Composites: Flame Retardancy, Mechanical Performance and Mechanisms. Nanotechnology.

[5] Wang Z., Liu J., Wu S., Wang W., Zhang L. (2010). Novel Percolation Phenomena and Mechanism of Strengthening Elastomers by Nanofillers. Phys. Chem. Chem. Phys..

[6] Lei Y., He J., Zhao Q., Liu T. (2018). A Nitrile Functionalized Graphene Filled Ethylene Propylene Diene Terpolymer Rubber Composites with Improved Heat Resistance. Compos. Part B Eng..

[7] Yuan Z., Yang Z., Ding Y., Li C., Ye L. (2019). Synergistic Reinforcing and Stabilizing Effect of Carbon Black-Zinc Dimethacrylate on the Ethylene Propylene Diene Monomer and Improving Mechanism of Sealing Resilience. Ind. Eng. Chem. Res..

[8] Dash B.K., Achary P.G.R., Nayak N.C., Choudhary R.N.P. (2017). Dielectric Relaxation Behavior of Exfoliated Graphite Nanoplatelet-Filled EPDM Vulcanizates. J. Electron. Mater..

[9] Mahapatra S.P., Sridhar V., Chaudhary R.N.P., Tripathy D.K. (2008). AC Conductivity and Positive Temperature Coefficient Effect in Microcellular EPDM Vulcanizates. Polym. Compos..

[10] Domenech S.C., Bendo L., Mattos D.J.S., Borges N.G., Zucolotto V., Mattoso L.H.C., Soldi V. (2009). Elastomeric Composites Based on Ethylene-Propylene-Diene Monomer Rubber and Conducting Polymer-Modified Carbon Black. Polym. Compos..

[11] Malas A., Hatui G., Pal P., Das C.K. (2014). Synergistic Effect of Expanded Graphite/Carbon Black on the Physical and Thermo-Mechanical Properties of Ethylene Propylene Diene Terpolymer. Polym.-Plast. Technol. Eng..

[12] Lee C.H., Kim S.W. (2000). Effects of Carbon Blacks on Electrical Properties of EPDM Compounds. J. Appl. Polym. Sci..

[13] Khaled M.A., Hassan E.A., Elwy A., Metwally E.E. (1994). Effect of Carbon Black on the Electrical and Creep Characteristics of EPDM Rubber. Mater. Lett..

[14] Hamza S.S. (1998). Effect of Aging and Carbon Black on the Mechanical Properties of EPDM Rubber. Polym. Test..

[15] Hu X., Gao H., Zhou X., Cui Y., Ge H. (2014). A New Approach to Rubber Reinforcement. RSC Adv..

[16] Dijkhuis K.A.J., Noordermeer J.W.M., Dierkes W.K. (2009). The Relationship between Crosslink System, Network Structure and Material Properties of Carbon Black Reinforced EPDM. Eur. Polym. J..

[17] Valentini L., Bolognini A., Alvino A., Bittolo Bon S., Martin-Gallego M., Lopez-Manchado M.A. (2014). Pyroshock Testing on Graphene Based EPDM Nanocomposites. Compos. Part B Eng..

[18] Rybiński P., Syrek B., Marzec A., Szadkowski B., Kuśmierek M., Śliwka-Kaszyńska M., Mirkhodjaev U.Z. (2021). Effects of Basalt and Carbon Fillers on Fire Hazard, Thermal, and Mechanical Properties of Epdm Rubber Composites. Materials.

[19] Lu S., Bai Y., Wang J., Chen D., Ma K., Meng Q., Liu X. (2019). Flexible GnPs/EPDM with Excellent Thermal Conductivity and Electromagnetic Interference Shielding Properties. Nano.

[20] Beutier C., David L., Sudre G., Cassagnau P., Heuillet P., Cantaloube B., Serghei A. (2022). In-Situ Coupled Mechanical/Electrical Investigations of EPDM/CB Composite Materials: The Electrical Signature of the Mechanical Mullins Effect. Compos. Sci. Technol..

[21] Jha N., Sarkhel G., Mahapatra S.P. (2019). Morphology, Barrier and Electrical Properties of Oil-Extended EPDM/Nanographite Nanocomposites. Mater. Today Proc..

[22] Araby S., Zaman I., Meng Q., Kawashima N., Michelmore A., Kuan H.C., Majewski P., Ma J., Zhang L. (2013). Melt Compounding with Graphene to Develop Functional, High-Performance Elastomers. Nanotechnology.

[23] Valentín J.L., Mora-Barrantes I., Carretero-González J., López-Manchado M.A., Sotta P., Long D.R., Saalwächter K. (2010). Novel Experimental Approach to Evaluate Filler-Elastomer Interactions. Macromolecules.

[24] Papageorgiou D.G., Kinloch I.A., Young R.J. (2015). Graphene/Elastomer Nanocomposites. Carbon N. Y..

[25] Watt M.R., Gerhardt R.A. (2020). Factors That Affect Network Formation in Carbon Nanotube Composites and Their Resultant Electrical Properties. J. Compos. Sci..

[26] Paszkiewicz S., Pypeć K., Irska I., Piesowicz E. (2020). Functional Polymer Hybrid Nanocomposites Based on Polyolefins: A Review. Processes.

[27] Babel V., Hiran B.L. (2021). A Review on Polyaniline Composites: Synthesis, Characterization, and Applications. Polym. Compos..

[28] Niyobuhungiro D., Hong L. (2021). Graphene Polymer Composites: Review on Fabrication Method, Properties and Future Perspectives. Adv. Sci. Technol. Res. J..

[29] Mensah B., Gupta K.C., Kang G., Lee H., Nah C. (2019). A Comparative Study on Vulcanization Behavior of Acrylonitrile-Butadiene Rubber Reinforced with Graphene Oxide and Reduced Graphene Oxide as Fillers. Polym. Test..

[30] Mensah B., Kumar D., Lim D.K., Kim S.G., Jeong B.H., Nah C. (2015). Preparation and Properties of Acrylonitrile-Butadiene Rubber-Graphene Nanocomposites. J. Appl. Polym. Sci..

[31] Giri S., Wan C. (2016). Electronic Applications of Ethylene Vinyl Acetate and Its Composites.

[32] Nwabunma D., Kyu T. (2007). Polyolefin Composites.

[33] Mensah B., Gupta K.C., Kim H., Wang W., Jeong K.U., Nah C. (2018). Graphene-Reinforced Elastomeric Nanocomposites: A Review. Polym. Test..

[34] Ke K., Yue L., Shao H., Yang M.B., Yang W., Manas-Zloczower I. (2021). Boosting Electrical and Piezoresistive Properties of Polymer Nanocomposites via Hybrid Carbon Fillers: A Review. Carbon N. Y..

[35] Szeluga U., Kumanek B., Trzebicka B. (2015). Synergy in Hybrid Polymer/Nanocarbon Composites. A Review. Compos. Part A Appl. Sci. Manuf..

[36] Safdari M., Al-haik M.S. (2018). A Review on Polymeric Nanocomposites: Effect of Hybridization and Synergy on Electrical Properties.

[37] Namasivayam M., Shapter J. (2017). Factors Affecting Carbon Nanotube Fillers towards Enhancement of Thermal Conductivity in Polymer Nanocomposites: A Review. J. Compos. Mater..

[38] Kumar V., Kalia S., Swart H.C., Kumar V., Kalia S., Swart H.C. (2017). Conducting Polymer Hybrids.

[39] Zhang H., Zhang G., Tang M., Zhou L., Li J., Fan X., Shi X., Qin J. (2018). Synergistic Effect of Carbon Nanotube and Graphene Nanoplates on the Mechanical, Electrical and Electromagnetic Interference Shielding Properties of Polymer Composites and Polymer Composite Foams. Chem. Eng. J..

[40] Jun Y.S., Um J.G., Jiang G., Yu A. (2018). A Study on the Effects of Graphene Nano-Platelets (GnPs) Sheet Sizes from a Few to Hundred Microns on the Thermal, Mechanical, and Electrical Properties of Polypropylene (PP)/GnPs Composites. Express Polym. Lett..

[41] Evgin T., Turgut A., Hamaoui G., Spitalsky Z., Horny N., Micusik M., Chirtoc M., Sarikanat M., Omastova M. (2020). Size Effects of Graphene Nanoplatelets on the Properties of High-Density Polyethylene Nanocomposites: Morphological, Thermal, Electrical, and Mechanical Characterization. Beilstein J. Nanotechnol..

[42] Araby S., Saber N., Ma X., Kawashima N., Kang H., Shen H., Zhang L., Xu J., Majewski P., Ma J. (2015). Implication of Multi-Walled Carbon Nanotubes on Polymer/Graphene Composites. Mater. Des..

[43] Su J., Li C. (2021). Effect of Plasma-Enhanced Chemical Vapor Deposition (PECVD) Graphene Content on the Properties of EPDM/Graphene Composites. J. Mater. Sci. Mater. Electron..

[44] Zakaria M.R., Abdul Kudus M.H., Akil H.M., Mohd Thirmizir M.Z. (2017). Comparative Study of Graphene Nanoparticle and Multiwall Carbon Nanotube Filled Epoxy Nanocomposites Based on Mechanical, Thermal and Dielectric Properties. Compos. Part B Eng..

[45] Zhang H., Wang C., Zhang Y. (2015). Preparation and Properties of Styrene-Butadiene Rubber Nanocomposites Blended with Carbon Black-Graphene Hybrid Filler. J. Appl. Polym. Sci..

[46] Cai J., Hu N., Wu L., Liu Y., Li Y., Ning H., Liu X., Lin L. (2019). Preparing Carbon Black/Graphene/PVDF-HFP Hybrid Composite Films of High Piezoelectricity for Energy Harvesting Technology. Compos. Part A Appl. Sci. Manuf..

[47] Xue C., Gao H., Hu G. (2020). Viscoelastic and Fatigue Properties of Graphene and Carbon Black Hybrid Structure Filled Natural Rubber Composites under Alternating Loading. Constr. Build. Mater..

[48] Mondal S., Khastgir D. (2017). Elastomer Reinforcement by Graphene Nanoplatelets and Synergistic Improvements of Electrical and Mechanical Properties of Composites by Hybrid Nano Fillers of Graphene-Carbon Black & Graphene-MWCNT. Compos. Part A Appl. Sci. Manuf..

[49] Wang L.-L., Tong Y.-P., Zhang L.-Q., Tian M. (2010). Comparison of Ethylene-Propylene Diene Terpolymer Composites Filled with Natural and Synthesized Micas. J. Appl. Polym. Sci..

[50] Frounchi M., Dadbin S., Salehpour Z., Noferesti M. (2006). Gas Barrier Properties of PP/EPDM Blend Nanocomposites. J. Membr. Sci..

