# Supplementary material for: Morphological, Mechanical and Gas Penetration Properties of Elastomer Composites with Hybrid Fillers
Source: Polymers (Basel). 2022 Sep 27;14(19):4043. doi: 10.3390/polym14194043 (PMC9572072; doi:10.3390/polym14194043)
Supplement: Supplementary file 1 [file polymers-14-04043-s001.zip › polymers-1807893-supplementary.pdf]

polymers

Supplementary Information

Morphological, Mechanical and Gas Penetration Properties of Elastomer Composites with Hybrid Fillers

Tuba Evgin [1,2,]*, Matej Mičušík [1], Peter Machata [1], Hamed Peidayesh [1], Jozef Preťo [3], and Mária Omastová [1*]

[1] Polymer Institute of the Slovak Academy of Sciences, 845 41 Bratislava, Slovakia
[2] Mechanical Engineering Department, Engineering Faculty, Dokuz Eylul University, Izmir 35397, Turkey
[3] Vipo, a.s., Generála Svobodu 1069/4, 958 01 Partizánske, Slovakia
* Correspondence: tuba.evgin@deu.edu.tr (T.E.); maria.omastova@savba.sk (M.O.)

1.1. X-ray Photoelectron Spectroscopy Analysis of Carbon-Based Materials

Table S1. Apparent surface chemical composition of CB as determined by XPS.

Sample	C1s	O1s	S2p
	**sp^2/sp^3/C-O/C=O/OC=O/π-π***	**$C{=}O_{ar}/OC{=}O^x/C{-}O/O_2C{=}O$**	**$C{-}S/SO_4$**
	**(284.3/284.8/286.0/287.3/289.2/290.9)**	**(531.6/532.2/533.5/5354.8)**	**(163.9/168.4)**
CB	**95.6**<br>62.2/23.3/4.1/0.6/2.1/3.6	**5.0**<br>0.4/1.4/2.2/-	**0.3**<br>0.2/0.1

Table S2. Apparent surface chemical composition of GnPs as determined by XPS.

Sample	C1s	O1s	S2p
	**sp^2/sp^3/C-O/C=O/OC=O/π-π***	**$C{=}O_{ar}/OC{=}O^x/C{-}O/O_2C{=}O$**	**$C{-}S/SO_3$**
	**(284.4/284.7/286.4/287.2/289.2/291.2)**	**(531.4/532.5/533.5/535.7)**	**(163.8/168.1)**
G1	**96.4**<br>84.1/1.8/0.5/0.7/1.3/8.0	**3.2**<br>1.4/0.6/0.2/0.9	**0.6**<br>0.4/0.2
G2	**96.5**<br>79.3/7.1/0.8/0.5/1.1/7.7	**3.0**<br>1.3/0.8/0.2/0.7	**0.6**<br>0.4/0.2
G3	**96.4**<br>84.3/1.7/0.3/0.8/1.2/8.0	**3.1**<br>1.1/1.1/0.9/0.1	**0.5**<br>0.2/0.3
G4	**95.3**<br>77.8/6.0/1.3/1.2/1.7/7.2	**4.7**<br>1.0/2.1/1.4/0.2	**0.1**<br>0/0.1

2.1. Mechanical Properties Results of EPDM-based Composites

Table S3. Mechanical properties of EPDM/GnPs composites.

Samples	GnPs (Phr)	Young's modul (MPa)	Strain at Break (%)	Stress at Tens.Strength (MPa)	Modul 100 (MPa)	Modul 300 (MPa)
Unfilled EPDM		1.61±0.16	362.91±67.07	1.53±0.16	0.95±0.05	1.38±0.07
EPDM/G1	1	1.73±0.06	350.74±71.25	1.58±0.23	0.91±0.02	1.43±0.04
	7	2.27±0.05	466.76±22.77	2.31±0.09	1.14±0.02	1.75±0.02
	10	2.95±0.32	360.32±93.76	2.43±0.29	1.37±0.10	2.17±0.11
	15	3.52±0.33	573.10±94.84	3.72±0.52	1.65±0.10	2.50±0.13
	20	4.12±0.16	612.22±60.99	4.15±0.38	1.87±0.04	2.77±0.06
EPDM/G2	1	1.67±0.19	318.39±37.18	1.58±0.08	0.96±0.06	1.49±0.03
	7	2.21±0.28	439.17±49.80	2.47±0.18	1.24±0.03	1.97±0.06
	10	2.78±0.11	483.94±12.03	2.69±0.14	1.33±0.05	2.05±0.07
	15	3.32±0.10	480.72±72.69	2.74±0.31	1.45±0.06	2.16±0.07
	20	3.63±0.19	579.31±59.22	3.40±0.51	1.64±0.11	2.38±0.17
EPDM/G3	1	1.74±0.06	296.53±40.00	1.50±0.13	0.96±0.05	1.52±0.09
	7	2.10±0.16	431.85±63.85	2.37±0.41	1.18±0.11	1.89±0.13
	10	2.56±0.21	437.22±93.61	2.45±0.41	1.26±0.06	1.97±0.07
	15	3.10±0.15	586.91±95.87	2.98±0.44	1.41±0.04	2.07±0.07
	20	3.79±0.21	553.52±49.07	3.42±0.27	1.67±0.04	2.45±0.03
EPDM/G4	1	1.58±0.22	308.47±86.79	1.48±0.27	0.90±0.05	1.41±0.02
	7	1.17±0.28	679.40±74.41	4.40±0.73	1.02±0.03	1.65±0.06
	10	1.32±0.11	756.62±40.73	6.10±0.71	0.93±0.05	1.68±0.05
	15	1.43±0.08	896.30±17.93	8.43±0.28	1.07±0.03	1.89±0.05
	20	0.87±0.02	897.22±46.64	6.38±0.31	0.97±0.01	1.73±0.04

Table S4. Mechanical properties of EPDM/CB/G1 composites.

Samples	GnPs (Phr)	Young's modul (MPa)	Strain at Break (%)	Stress at Tens.Strength (MPa)	Modul 100 (MPa)	Modul 300 (MPa)
Unfilled EPDM		1.61±0.16	362.91±67.07	1.53±0.16	0.95±0.05	1.38±0.07
EPDM/CB 20 phr/G1	0	1.82±0.45	531.11±80.18	7.23±1.37	1.64±0.14	3.77±0.25
	1	1.88±0.32	471.11±54.10	6.64±0.92	1.64±0.09	3.99±0.17
	3	2.02±0.18	748.93±57.73	10.79±1.15	1.59±0.14	3.63±0.19
	5	1.88±0.16	735.35±37.76	10.32±0.82	1.68±0.06	3.73±0.06
	7	2.05±0.31	629.63±86.76	9.10±1.82	1.92±0.13	4.23±0.33
EPDM/CB 50 phr/G1	0	2.88±0.34	815.22±56.65	17.55±2.36	2.28±0.17	6.10±0.39
	1	2.64±0.21	751.00±67.00	16.00±1.80	2.44±0.04	6.29±0.10
	3	2.72±0.26	707.39±73.67	16.77±2.38	2.68±0.11	7.09±0.26
	5	3.18±0.13	764.72±32.98	20.59±1.04	3.02±0.07	8.00±0.27
	7	2.88±0.17	719.17±92.43	18.51±2.53	3.14±0.08	7.98±0.18

3.1. Dynamic Mechanical Analysis of EPDM-based Composites

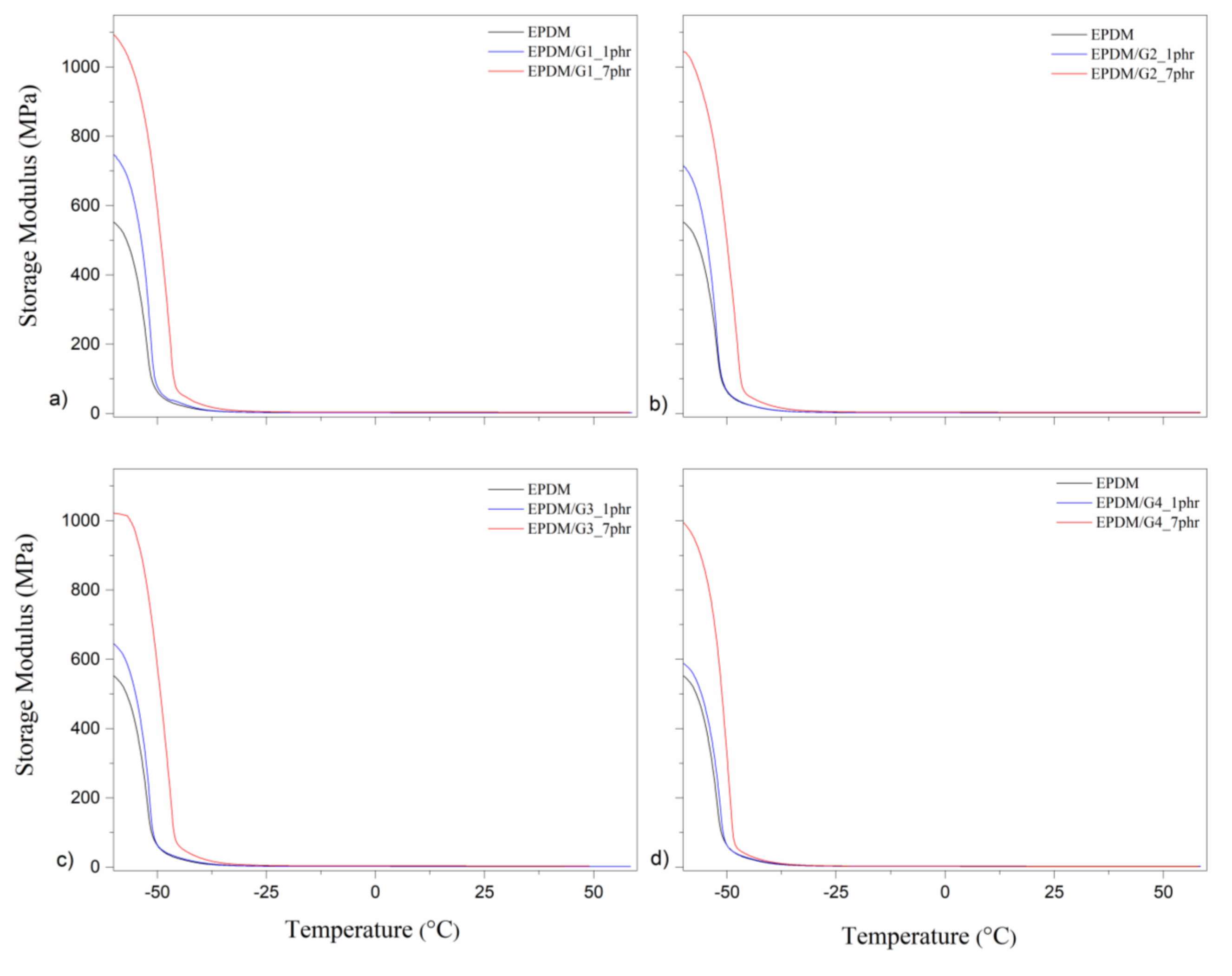

Figure S1. Storage modulus (E′) for a) EPDM/G1, b) EPDM/G2, c) EPDM/G3, and d) EPDM/G4 composites.

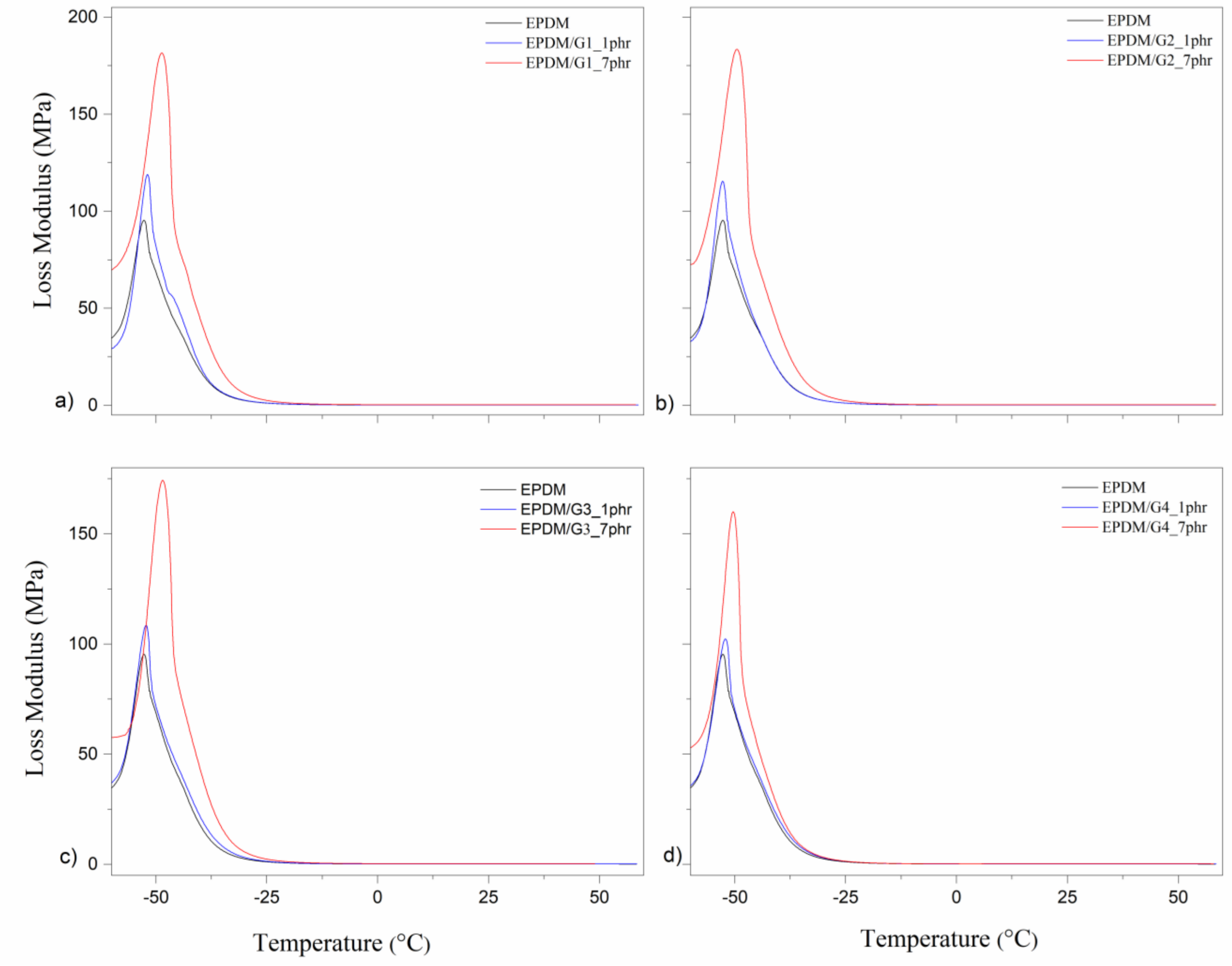

Figure S2. Loss modulus for a) EPDM/G1, b) EPDM/G2, c) EPDM/G3, and d) EPDM/G4 composites.

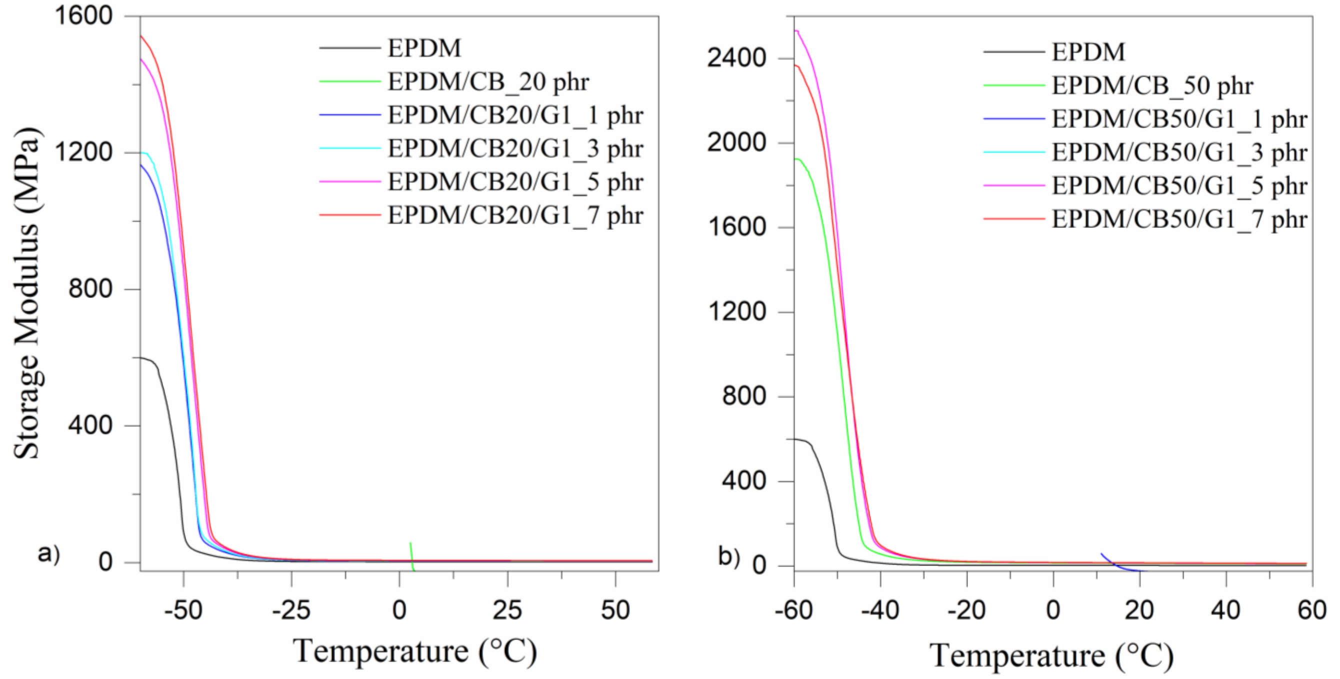

Figure S3. Storage modulus (E′) for a) EPDM/CB20/G1 and b) EPDM/CB50/G1 composites.

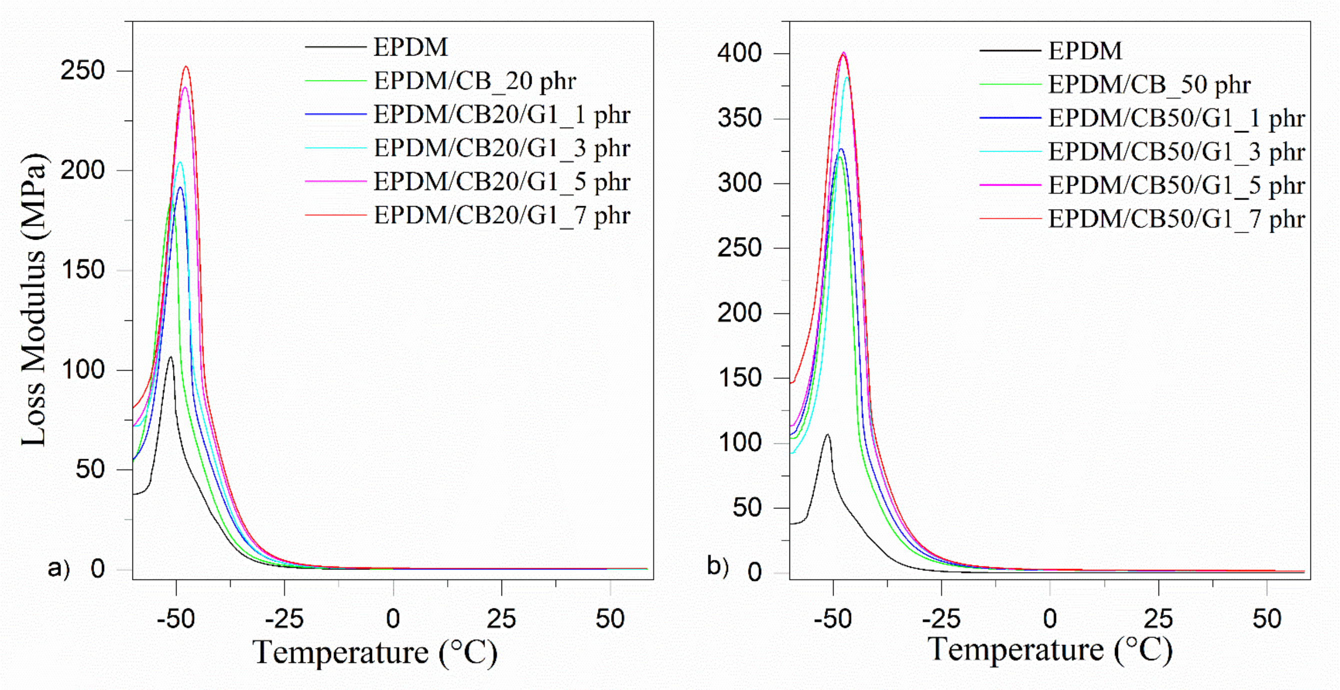

Figure S4. Loss modulus for a) EPDM/CB20/G1 and b) EPDM/CB50/G1 composites.
